# The Effect of Microbiota and the Immune System on the Development and Organization of the Enteric Nervous System

**DOI:** 10.1053/j.gastro.2016.07.044

**Published:** 2016-11

**Authors:** Yuuki Obata, Vassilis Pachnis

**Affiliations:** The Francis Crick Institute, London, United Kingdom

**Keywords:** Enteric Nervous System (ENS), Microbiota, Neuroimmune Interaction, Parkinson’s Disease, Microbiota–Gut–Brain Axis, BMP, bone morphogenetic protein, BSH, bile salt hydrolase, CNS, central nervous system, EC, enterochromaffin cell, EGC, enteric glial cell, ENS, enteric nervous system, GF, germ-free, GLP-1, glucagon-like peptide-1, GI, gastrointestinal, 5-HT, 5-hydroxytryptamine, IBS, irritable bowel syndrome, MCT, monocarboxylate transporter, MGB, microbiota–gut–brain, MM, muscularis macrophage, nNOS, neuronal nitric oxide synthase, PD, Parkinson’s disease, RSD, resistant starch diet, SCFA, short-chain fatty acid, SERT, serotonin-selective reuptake transporter, TLR, Toll-like receptor

## Abstract

The gastrointestinal (GI) tract is essential for the absorption of nutrients, induction of mucosal and systemic immune responses, and maintenance of a healthy gut microbiota. Key aspects of gastrointestinal physiology are controlled by the enteric nervous system (ENS), which is composed of neurons and glial cells. The ENS is exposed to and interacts with the outer (microbiota, metabolites, and nutrients) and inner (immune cells and stromal cells) microenvironment of the gut. Although the cellular blueprint of the ENS is mostly in place by birth, the functional maturation of intestinal neural networks is completed within the microenvironment of the postnatal gut, under the influence of gut microbiota and the mucosal immune system. Recent studies have shown the importance of molecular interactions among microbiota, enteric neurons, and immune cells for GI homeostasis. In addition to its role in GI physiology, the ENS has been associated with the pathogenesis of neurodegenerative disorders, such as Parkinson’s disease, raising the possibility that microbiota–ENS interactions could offer a viable strategy for influencing the course of brain diseases. Here, we discuss recent advances on the role of microbiota and the immune system on the development and homeostasis of the ENS, a key relay station along the gut–brain axis.

The gastrointestinal (GI) tract is essential for digestion of foods, absorption of nutrients and water, energy balance, and protection from pathogenic microorganisms. Most aspects of GI physiology are under neural control, which is exerted via extrinsic nerves (which include both primary afferent and autonomic fibers that ultimately connect the gut tissues with the central nervous system [CNS]) and a vast network of intrinsic enteric neurons (1–5 × 10^8^) and glial cells that are organized into the myenteric and submucosal plexi of the enteric nervous system (ENS).^1^ On the basis of their neurochemical properties, enteric neurons are subdivided into multiple subtypes that share molecular, morphologic, and physiological characteristics.^2^, ^3^ Unlike enteric neurons whose cell bodies are restricted to the myenteric and submucosal ganglia, enteric glial cells (EGCs), and neuronal fibers are distributed throughout the gut wall, including the lamina propria of the mucosa.^4^, ^5^ Among other functions, the ENS regulates GI peristalsis, epithelial secretion, intestinal blood flow, and transmucosal movement of liquids largely independently of central nervous system input.^1^

In addition to its motor and secretory functions, the GI tract is the largest sensory organ of the body, which incessantly monitors the dynamic microenvironment of the gut wall and its lumen.^6^ Several cellular systems contribute to the sensory function of the gut, including the enteroendocrine cells of the intestinal epithelium, the mucosal immune system, and the ENS.^1^ The integrated responses of these cellular networks enable the gut to build highly selective anatomic and functional barriers that allow absorption of useful nutrients and exclusion of harmful chemicals and micro-organisms. Information relating to the chemical composition and caloric value of ingested food, the dynamic equilibrium of the microbial ecosystem of the gut (microbiota), and the physiological state of the gut wall reaches the brain via the neurohumoral pathways of the microbiota–gut–brain (MGB) axis and allows the CNS to generate appropriate homeostatic and behavioral responses.^6^, ^7^, ^8^

Emerging evidence suggests that gut microflora can have dramatic effects on the development and function of the nervous system, both at the local as well as at the systemic level. Although disruption of the physiological microbiota composition (dysbiosis) is known to influence cognitive activity and behavior, such as stress response, anxiety, and memory,^7^, ^9^ the mechanistic understanding of microbe–neural interactions remains obscure. Because the ENS constitutes a key relay station along the MGB axis, understanding the mechanisms of ENS–microbe communication is essential for deciphering how the gut microenvironment influences physiology at the local and organismal level. Here, we provide a brief overview of the impact of microbiota and the mucosal immune system on ENS development and homeostasis.

## Genetic Programs That Control ENS Development

The ENS is derived from neuroectodermal progenitors that originate mainly from the vagal neural crest, invade the foregut during embryogenesis, and migrate rostrocaudaly to colonize the entire GI tract. In mammals, enteric neurogenesis and gliogenesis occur mostly during embryonic and fetal stages but a considerable fraction of enteric neurons and glia are born within the postnatal gut.^10^ Furthermore, functional maturation of intestinal neural circuits also occurs within the early postnatal period.^11^ To date, the development of the ENS has been examined primarily from the point of view of genetic and molecular mechanisms that operate within the neuroectodermal lineages of the gut. These studies have identified several transcription factors, such as SOX10 (an SRY-related HMG-box transcription factor), FOXD3 (a member of the forkhead protein family) and HAND2,^12^, ^13^, ^14^, ^15^ which control the survival and lineage choices of ENS progenitors. In addition, ENS lineages express several types of cell surface receptors, such as the receptor tyrosine kinase RET and the G-protein–coupled endothelin receptor B (EDNRB), which control multiple aspects of ENS development and neural circuit assembly. RET and EDNRB are activated by members of the GDNF family of ligands and endothelin-3, respectively, which are produced by the intestinal mesenchyme, highlighting the key role of the cellular microenvironment on the development of ENS lineages.^16^ For a comprehensive recent review of the cellular and molecular mechanisms underlying ENS development, please refer to Lake et al.^16^

## The Role of Gut Microbial Factors on the Development and Homeostasis of the ENS

Immediately after birth the GI tract is colonized by complex microbial communities (>100 trillion microbes belonging to ∼1000 species), which influence multiple aspects of host physiology, including metabolism, immune responses, behavior, and circadian rhythm.^7^, ^17^, ^18^, ^19^ The role of microbiota on ENS organization is highlighted by the reduced number of enteric neurons and the associated deficits in gut motility observed in germ-free (GF) mice.^20^, ^21^, ^22^ In addition, GF mice show attenuated excitability of intrinsic primary afferent neurons^23^ that are part of the hard-wired gut-brain neural pathways.^24^ Furthermore, the development and continuous homeostatic influx of EGCs into the intestinal mucosa is defective in GF mice.^25^ These observations argue that microbiota is essential for the assembly of intestinal neural circuits and for signaling along the gut–brain axis. Interestingly, reconstitution of GF mice with conventional microbiota normalized the density of EGC network and gut physiology,^25^, ^26^ raising interesting questions relating to the cellular plasticity of the ENS and the mechanisms by which microbiota influence its homeostasis.

A recent report showed that the reduction of myenteric neurons in GF mice is present by postnatal day 3^27^ when the number and diversity of gut microbiota has not been established,^28^ raising the possibility that, in addition to factors associated with changes in early postnatal gut physiology or feeding, maternal microbial factors may play a role in ENS development during pregnancy via uteroplacental circulation. Consistent with this idea, microbial colonization of the gut may occur before birth.^29^, ^30^ Furthermore, recent evidence indicates that maternal microbe-derived factors and the maternal immune system contribute to the offspring’s immune and neuronal homeostasis.^31^, ^32^, ^33^, ^34^ Taken together, these observations suggest that dynamic host–microbe interactions during critical developmental periods could increase the risk of neurodevelopmental disorders and have long-term consequences on neuronal function. Here, we summarize the impact of gut microbial factors on ENS development and homeostasis.

### Toll-Like–Receptor Ligands

Gut microbe-derived signals are recognized partly by pattern recognition receptors, such as Toll-like receptors (TLRs). TLR4-/- mice are characterized by abnormal intestinal motility and a reduced number of nitrergic (neuronal nitric oxide synthase [nNOS]^+^) inhibitory neurons, a phenotype similar to that observed in GF and antibiotic-treated animals.^22^ This phenotype also was reproduced in mice with ENS-specific deletion of MyD88, an adaptor molecule essential for TLR-mediated signal transduction, suggesting key roles of the TLR4 pathway on the development and functional organization of intestinal neural networks.^22^ A separate study showed that deletion of TLR2, which is expressed by enteric neurons, EGCs, and smooth muscle cells of the gut wall, also resulted in reduction of nNOS^+^ neurons and acetylcholine-esterase–stained fibers in the myenteric ganglia.^35^ The altered neurochemical profiles of enteric neurons in the gut of TLR2-deficient mice was accompanied by gut dysmotility and attenuated chloride production by intestinal explants. Interestingly, expression of glial markers, such as glial fibrillary acidic protein (GFAP) and S100β, also decreased in the myenteric plexus of mutant mice. Considering that probiotic and pathogenic bacteria up-regulate TLR2 expression on human EGCs,^36^ these studies suggest that the microbiota–TLR2 pathway promotes functional maturation of EGCs. Interestingly, expression of GDNF is reduced significantly in TLR2-deficient and microbiota-depleted mice, while administration of GDNF rescued the ENS deficits of these animals, suggesting that the effects of TLR/microbiota pathways on ENS development and homeostasis are mediated via mesenchyme-derived neurotrophic factors. Outside the gut, TLR4 regulates the expression of *Sox10* and *Foxd3*, raising the possibility that these transcriptional regulators also are targets of TLR4 in the ENS.^37^, ^38^ Nevertheless, the molecular mechanisms by which TLR ligands control enteric neurogenesis during neonatal stages require further investigation.

### Short-Chain Fatty Acids

Gut microbes metabolize dietary fiber and resistant starch to produce a wide variety of metabolites, including short-chain fatty acids (SCFAs), which are used as nutrients by colonic epithelial cells and influence host physiology.^39^, ^40^, ^41^, ^42^ Recent studies have uncovered a role of SCFAs in the production of serotonin (5-hydroxytryptamine [5-HT]) by enterochromaffin cells (ECs) of the intestinal epithelium.^43^, ^44^ ECs are the largest source of serotonin in the body, which, among other functions, regulate GI motility and platelet function.^45^, ^46^ Spore-forming bacteria from healthy human beings and mouse microbiota increase colonic and serum 5-HT levels in GF mice (by increasing expression of the colonic biosynthetic enzyme tryptophan hydroxylase 1-Tph1^43^) and ameliorate GF-associated gut dysmotility. These bacteria produce SCFAs,^39^, ^47^ which are capable of increasing 5-HT production by cultured chromaffin cells^43^ and up-regulating Tph1 expression in a human-derived EC cell line.^44^ In addition, the extracellular availability of 5-HT within the gut is regulated by the serotonin-selective reuptake transporter (SERT) which is expressed by intestinal epithelial cells.^48^ Expression of SERT is lower in neonatal gut in comparison with adult tissues, resulting in higher availability of 5-HT, which during these early stages is essential for the maturation of intestinal motor reflexes.^48^, ^49^ SERT expression by intestinal epithelial cells also is regulated by microbiota-derived factors such as TLR ligands because treatment of an epithelial cell line (Caco-2) with lipopolysaccharide diminishes the expression and activity of SERT.^50^ These observations suggest that different kinds of microbial factors (eg, SCFAs and lipopolysaccharide) contribute to functional maturation of ENS by regulating the production and availability of 5-HT in a coordinated way.

SCFAs also can influence the neurochemical phenotype of the ENS in adult rat.^51^ Resistant starch diet (RSD), which enhances luminal SCFA concentration,^52^ specifically increased the proportion of excitatory cholinergic neurons in the colon, but had no effect on nNOS^+^ neurons, resulting in decreased colonic transit time. Intrarectal administration of butyrate, but not acetate or propionate, mimicked the effect of RSD. Of note, the butyrate-induced increase in excitatory choline acetyltransferase (ChAT)+ neurons depended on the butyrate transporter monocarboxylate transporter (MCT)2, which is expressed by enteric neurons,^51^ but the factors regulating neuronal MCT2 expression remain unknown. A recent study has shown that the EGC cell line, JUG-2, also expresses MCT1 and MCT2,^53^ although the physiological role of these enzymes on glial homeostasis in vivo has not been determined.

SCFAs also activate G-protein–coupled receptors, such as GPR41 and GPR43.^54^, ^55^ Analysis of transgenic reporter mice has shown that GPR41 is expressed by ECs and enteric neurons,^55^ although the role of neuronal GPR41 on ENS function has not been characterized. GPR43 is expressed by intestinal immune cells and sporadically by ECs.^55^ Activation of GPR43 on enteroendocrine cells by SCFAs promotes secretion of the incretin hormone glucagon-like peptide-1 (GLP-1),^56^ which also controls gastric emptying and gastrointestinal transit.^57^ Moreover, RSD is known to increase GLP-1 expression by cecal and colonic epithelial cells in vivo,^58^ suggesting that gut microbiota may increase GLP-1 levels through the SCFAs/GPR43/GPR41 pathway. An additional pathway inducing GLP-1 by microbiota is mediated by intestinal *Escherichia coli*–derived protein caseinolytic protease B. Caseinolytic protease B serves as an antigen mimetic of the α-melanocyte–stimulating hormone^59^ and is capable of stimulating melanocortin receptor 4 on enteroendocrine L cells to produce GLP-1.^60^ However, a recent study showed that GF mice (which lack colonic SCFAs) show significantly higher levels of GLP-1 in the plasma, while colonization of GF mice with microbiota or treatment with SCFAs reduced GLP-1 expression in the colon.^61^ Interestingly, blocking the GLP-1 signaling with exendin 9-39 (Ex-9) (GLP-1 antagonist) completely rescued the slow intestinal transit observed in GF mice, and antibiotic-dependent reduction of intestinal transit was rescued by deletion of GLP-1, indicating that GLP-1 signaling is required to slow intestinal transit in GF conditions. Perhaps slower intestinal transit provides extra time for nutrient absorption during insufficient colonic energy availability (lack of SCFAs) under GF conditions. Further studies will be necessary to determine how SCFAs regulate the levels of GLP-1 and how GLP-1 controls the activity of intestinal neural circuits.

An alternative mechanism by which SCFAs could affect host gene expression is the inhibition of histone deacetylase activity.^62^, ^63^ Histone deacetylase inhibition enhances histone acetylation of gene regulatory elements and increases gene transcription.^64^ The epigenetic regulation of the immune system by gut microbial butyrate has been shown in colonic T cells^39^, ^65^ and macrophages.^66^ Although little is known about the epigenetic modifications of ENS by SCFAs, butyrate treatment enhances acetylation of the H3K9 in primary cultured enteric neurons and the EGC cell line JUG-2.^51^, ^53^ It would be interesting to determine the extent to which microbiota-derived SCFAs modulate the epigenetic status of genes and its role in enteric neurogenesis and gliogenesis.

### Bile Acid Metabolism and Dietary Factors

Gut microbes also participate in the conversion of primary bile acids synthesized de novo in the liver into secondary bile acids.^17^ Secondary bile acids can activate the G-protein–coupled bile acid receptor 1, which is known as TGR5. TGR5 is highly expressed in enteric neurons and enteroendocrine L cells^67^ and TGR5-deficient mice showed delayed colonic transit and reduced defecation frequency relative to wild-type mice.^68^ In addition, stimulation with TGR5 agonists induced colonic peristalsis in wild-type but not TGR5-deficient mice, suggesting the important role of TGR-5 signaling on intestinal propulsive activity. TGR5-dependent enhancement of peristalsis could be mediated partly by production of 5-HT, because stimulation of isolated distal colon with bile acids increased 5-HT production.^68^ Although a better mechanistic understanding is required, targeting of TGR5 emerges as a potential therapeutic strategy to alleviate symptoms of constipation and diarrhea.

**Figure 1:**
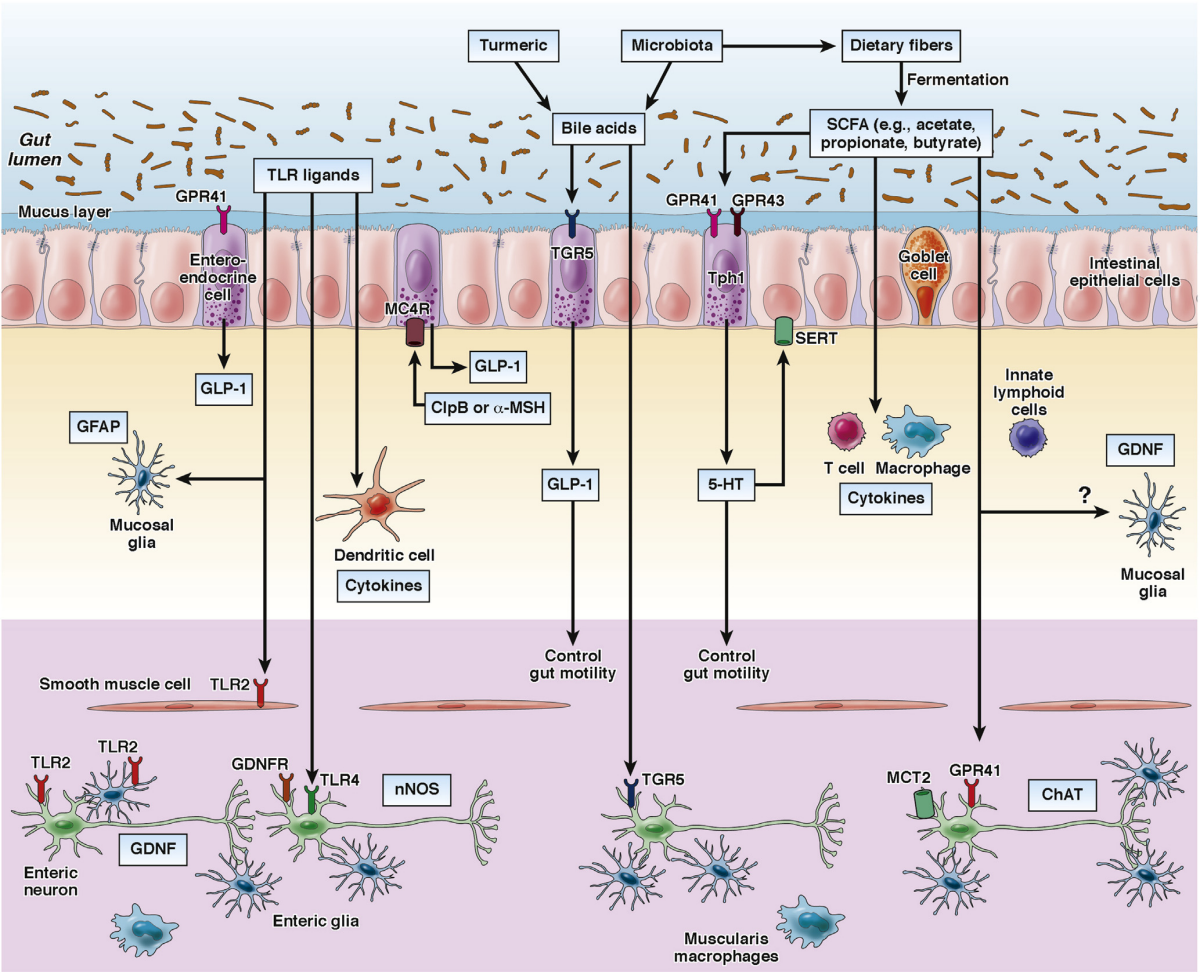
Microbiota and diet control the activity of multiple cell types in the gut wall, including the ENS. For example, the bacterial metabolites SCFAs activate G-protein coupled receptors (eg, GPR41 and GPR43) on enteroendocrine cells of the intestinal epithelium resulting in enhanced production of GLP-1 and 5-HT and changes in gut motility. Gut microbiota also contribute to the conversion of primary bile acids into secondary bile acids, which activate TGR5 expressed by enteroendocrine cells and enteric neurons. TLR signalling (eg, TLR2 and TLR4) maintains subsets of enteric neurons and influences gut motility. In addition, microbiota is essential for the maintenance of mucosal glial cells, which express the neurotrophic factor GDNF and GFAP. 5-HT, Serotonin; α-MSH, α-melanocyte-stimulating hormone; GDNF, glial cell-derived neurotrophic factor; GFAP, glial fibrillary acidic protein; GLP-1, glucagon-like peptide-1; SERT, serotonin-selective reuptake transporter; Tph1, tryptophan hydroxylase 1.

Diet ingredients also can influence gut motility and ENS function in combination with microbiota. A recent study identified deconjugated bile acids as fecal metabolites associated with intestinal transit time phenotype.^69^ Consumption of a Bangladeshi diet containing turmeric, a spice that increases luminal bile acids, significantly slowed motility in mice that had been colonized with microbiota isolated from a Bangladeshi donor, compared with a turmeric-free Bangladeshi diet, suggesting that a single food ingredient can influence gut motility. Because deconjugation of bile acids is mediated by activity of bacterial bile salt hydrolases (BSHs), these investigators generated gnotobiotic mice composed of either BSH-positive or BSH-negative bacterial consortia cultured from the microbiota of a Bangladeshi donor. Intestinal transit time significantly decreased in mice colonized with the BSH-positive microbiota than BSH-negative microbiota only when they were fed a turmeric-containing diet, indicating that the motility phenotypes are dependent on the abilities of microbiota to deconjugate the bile acids produced by turmeric consumption. Interestingly, this phenotype was not observed in mice heterozygous for a Ret null mutation, suggesting that the effects of turmeric on motility phenotypes depend on both genetic background and bacterial bile acid metabolism.^69^ Taken together, gut motility is influenced in a coordinated manner by the interaction between luminal environmental factors (eg, diet, SCFAs, bile acids, TLR ligands), microbial factors (composition and metabolism activity), and host factors (nutritional condition and functional ENS) (Figure 1).

## Bidirectional Communication Between the Gut Immune System and ENS

The GI tract harbors the highest concentration of immune cells in the body. In particular, macrophages, which have key roles in innate immune responses and tissue homeostasis, are present in intestinal muscularis (called *muscularis macrophages* [MMs]) and are in close contact with ENS cells.^70^ MMs have a unique surface marker profile (CX3CR1^hi^MHCII^hi^CD11c^lo^CD103^-^CD11b^+^) and their development is dependent on colony stimulatory factor (CSF)1 receptor, a receptor for macrophage CSF that regulates mononuclear phagocyte development.^71^ Interestingly, treatment of adult mice with the anti-CSF1R antibody induced depletion of MMs and resulted in gut dysmotility, manifested as colonic hyperactivity and increased colonic transit time. Bone marrow chimeric mice with *Csf1*-receptor–deficient hematopoietic progenitors also showed increased colonic transit time, indicating the importance of MMs on the physiological control of GI motility. MM-dependent control of gut motility is mediated by bone morphogenetic protein (BMP)2, which is expressed by MMs and activates enteric neurons.^72^, ^73^ Administration of the BMP signaling inhibitor dorsomorphin reproduced the phenotype of MM-depleted mice whereas exogenous BMP2 rescued the dysmotility of these animals. Remarkably, enteric neurons selectively express BMP receptor type II, a component of the BMP receptor, and can produce CSF1 that is required for MM development. CSF1-deficient mice harbored an increased number of enteric neurons and showed a less-organized ENS architecture. These results suggest that neuronal CSF1 contributes to the homeostasis of MMs, which then are required for normal ENS activity. Interestingly, production of CSF1 and BMP2 is dependent on gut microflora because antibiotic treatment decreased the production of both signaling mediators.^71^

More recently, Gabanyi et al^74^ performed RNA sequencing–based transcriptome analysis of MMs and lamina propria macrophages, and showed that MMs preferentially express tissue-protective and wound-healing genes resembling alternatively activated (M2-type) macrophages, while lamina propria macrophages express proinflammatory genes. Interestingly, MMs express *Adrb2* (encoding β2 adrenergic receptors), which is essential for norepinephrine signaling, and reside in close proximity to enteric neurons labeled with the calcium indicator GCaMP3, suggesting that MMs interact with active neurons in gut muscularis. Of note, intestinal infection with a mutant strain of *Salmonella typhimurium* activated tyrosine hydroxylase–expressing extrinsic neurons in the sympathetic ganglia innervating the gut, leading to production of norepinephrine in the muscular layer, which was accompanied by a significant increase in intestinal transit time. The noradrenaline signaling on MMs through β2 adrenergic receptors also contributed to their polarization into M2-type–related phenotype. These studies indicate that microbiota-driven interactions between innate immune cells and the ENS control gut motility and enhance the tissue-protective phenotype in MMs in response to intestinal infection, even in sites distal from an initial pathogen entry.^74^

**Figure 2:**
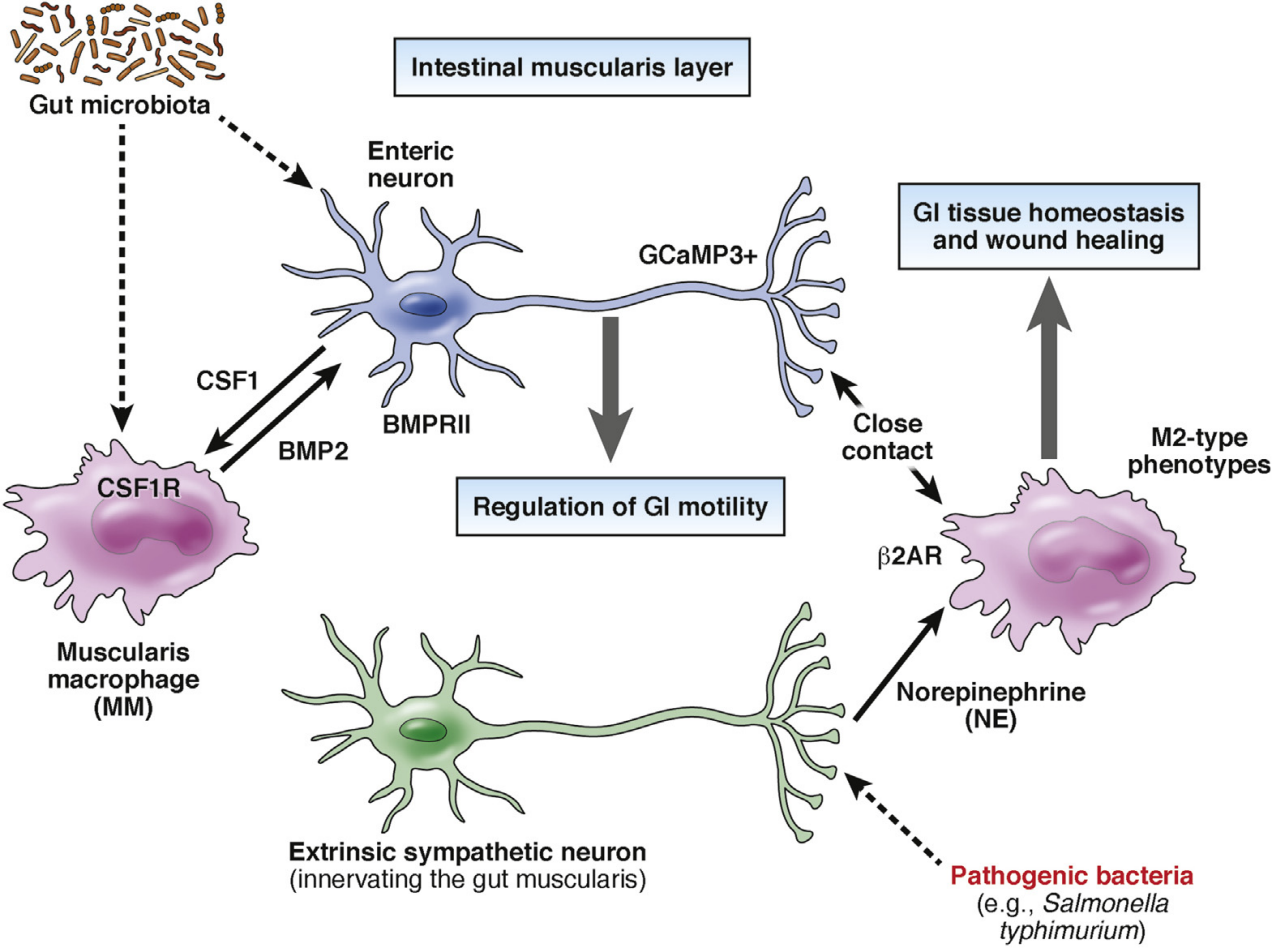
BMP2 from muscularis macrophages (MMs) regulates the activity of enteric neurons (by activating BMPRII) while CSF1 from enteric neurons is essential for the development of MMs (which express CSF1R). Production of CSF1 and BMP2 is dependent on gut microbiota. Activation of MMs by norepinephrine (via β2 adrenergic receptors) contributes to their polarization into M2-type phenotype, which is associated with tissue homeostasis and wound healing. BMP2, bone morphogenetic protein 2; β2AR: β2 adrenergic receptors, CSF1, colony stimulating factor 1; NE, norepinephrine.

In conclusion, these recent studies provide further support for the concept that specialized interactions between the ENS and the gut immune system are essential for GI tract homeostasis (Figure 2). Given the capacity of the nervous system to respond rapidly to diverse stimuli by releasing neurotransmitters and neuropeptides, which include among their targets immune cell functions,^75^ it is conceivable that enteric neural reflexes are an integral part of early response mechanisms that operate continuously to restore the balance between innocuous and pathogenic micro-organisms and thus maintain the symbiotic host–microbe relationships.

## Parkinson’s Disease: A Disease of the Microbiota–Gut–Brain Axis?

The critical role of the ENS in controlling GI tract physiology is highlighted by the high morbidity of congenital enteric neuron deficits, such as Hirschsprung’s disease.^76^ Acquired dysmotility syndromes, such as irritable bowel syndrome (IBS), also are attributed to ENS deficits, although additional local factors, including luminal microbiota, mucosal immune cells, epithelial barrier functions, and serotonin metabolism have been implicated in the pathogenesis of this condition.^77^ IBS also is associated with deficits in the bidirectional gut–brain communication,^78^ and studies on this condition are likely to provide insight into the role on the MGB axis in health and disease. A detailed presentation of the relationship between IBS and the MGB axis is beyond the scope of this review and the reader is directed to excellent recent literature.^77^, ^79^ Here, we highlight an emerging hypothesis implicating ENS deficits in the pathogenesis of neurodegenerative diseases, including Parkinson’s disease (PD).^80^ PD is characterized by selective degeneration of dopaminergic neurons in the midbrain substantia nigra, and the abnormal deposition of α-synuclein (Lewy bodies) in the surviving dopaminergic neurons, resulting in characteristic motor symptoms.^81^ However, a high percentage of PD patients also are characterized by abnormal GI motility and constipation.^82^ Interestingly, PD-associated α-synuclein accumulations also are found in enteric neurons, which precede the development of motor symptoms by several years,^83^ suggesting that the ENS is an initial site of α-synuclein aggregations, which subsequently spread to the brain through vagus nerve fibers. In support of this notion, the risk of PD is lower in vagotomized individuals in comparison with the healthy population.^84^ Furthermore, a recent study has shown that α-synuclein injected into the gut wall can translocate into the dorsal motor nucleus of the vagus nerve via vagus nerve fibers in a time-dependent manner.^85^ Nevertheless, it remains unclear whether in PD patients intestinal PD pathology spreads to the brain and initiates motor symptoms, and how luminal factors (eg, microbiota and diet) could influence the potential gut–brain translocation, severity of intestinal symptoms, and loss of midbrain dopaminergic neurons. Interestingly, PD patients show dysbiosis, which is correlated with the clinical phenotype,^86^ although it has not been determined whether the observed changes of microbiota contribute to the pathogenesis of the disease or instead are the consequence of PD-associated nonmotor symptoms. The other alterations found in the gut of PD patients was an abnormal increase of proinflammatory cytokine genes and the glial cell markers GFAP, SOX10, and S100β,^87^ suggesting an association of intestinal inflammation and glial dysregulation with PD development. In addition, colonic biopsy specimens from PD patients showed the presence of enteric glial reactivity characterized by the up-regulation of GFAP expression but a reduction in phosphorylation,^88^ although the pathophysiological significance of these abnormalities remains unknown. Given that PD patients are diagnosed only after the onset of motor symptoms and are not treated until significant loss of dopaminergic neurons already has occurred, intestinal PD pathology could be an early and potentially useful biomarker for this condition.

## Conclusions and Future Perspectives

Accumulating evidence suggests that the development and function of ENS is controlled by luminal microbial factors and the host immune system. In addition to the importance of ENS on GI homeostasis, ENS also serves as a relay station along the MGB axis that conveys information from the luminal microenvironment to the CNS. The mechanisms underlying MGB axis communication involve the immune and endocrine system, neural pathways via the vagus nerve, and the microbiota-dependent modulation of CNS.^8^, ^89^, ^90^, ^91^, ^92^, ^93^, ^94^, ^95^ For instance, SCFAs produced by microbiota ensure the integrity of the blood-brain barrier by up-regulating tight junction proteins,^42^ and regulate the maturation and activation of microglial cells.^41^ On the other hand, the mechanism directly controlling the neural pathway connecting the CNS and ENS by microbial factors remains elusive. Considering that defects in ENS cause the development of CNS diseases, understanding the molecular mechanism of microbiota–ENS interactions could help us generate novel therapeutic strategies for multiple types of neurodegenerative diseases.

## References

[1] Furness J.B., Furness J.B. (2007). The enteric nervous system.

[2] Lomax A.E., Furness J.B. (2000). Neurochemical classification of enteric neurons in the guinea-pig distal colon. Cell Tissue Res.

[3] Qu Z.-D., Thacker M., Castelucci P. (2008). Immunohistochemical analysis of neuron types in the mouse small intestine. Cell Tissue Res.

[4] Boesmans W., Lasrado R., Vanden Berghe P. (2014). Heterogeneity and phenotypic plasticity of glial cells in the mammalian enteric nervous system. Glia.

[5] Gulbransen B.D., Sharkey K.A. (2012). Novel functional roles for enteric glia in the gastrointestinal tract. Nat Rev Gastroenterol Hepatol.

[6] Williams E.K., Chang R.B., Strochlic D.E. (2016). Sensory neurons that detect stretch and nutrients in the digestive system. Cell.

[7] Cryan J.F., Dinan T.G. (2012). Mind-altering microorganisms: the impact of the gut microbiota on brain and behaviour. Nat Rev Neurosci.

[8] Collins S.M., Surette M., Bercik P. (2012). The interplay between the intestinal microbiota and the brain. Nat Rev Microbiol.

[9] Diaz Heijtz R., Heijtz R.D., Wang S. (2011). Normal gut microbiota modulates brain development and behavior. Proc Natl Acad Sci U S A.

[10] Laranjeira C., Sandgren K., Kessaris N. (2011). Glial cells in the mouse enteric nervous system can undergo neurogenesis in response to injury. J Clin Invest.

[11] Roberts R.R., Roberts R.R., Murphy J.F. (2006). Development of colonic motility in the neonatal mouse-studies using spatiotemporal maps. Am J Physiol Gastrointest Liver Physiol.

[12] Southard-Smith E.M., Southard-Smith E.M., Kos L. (1998). SOX10 mutation disrupts neural crest development in Dom Hirschsprung mouse model. Nat Genet.

[13] Mundell N.A., Plank J.L., LeGrone A.W. (2012). Enteric nervous system specific deletion of Foxd3 disrupts glial cell differentiation and activates compensatory enteric progenitors. Dev Biol.

[14] D'Autréaux F., Morikawa Y., Cserjesi P. (2007). Hand2 is necessary for terminal differentiation of enteric neurons from crest-derived precursors but not for their migration into the gut or for formation of glia. Development.

[15] Hendershot T.J., Liu H., Sarkar A.A. (2007). Expression of Hand2 is sufficient for neurogenesis and cell type-specific gene expression in the enteric nervous system. Dev Dyn.

[16] Lake J.I., Lake J.I., Heuckeroth R.O. (2013). Enteric nervous system development: migration, differentiation, and disease. Am J Physiol Gastrointest Liver Physiol.

[17] Tremaroli V., Bäckhed F. (2012). Functional interactions between the gut microbiota and host metabolism. Nature.

[18] Leone V., Gibbons S.M., Martinez K. (2015). Effects of diurnal variation of gut microbes and high-fat feeding on host circadian clock function and metabolism. Cell Host Microbe.

[19] Lee W.-J., Hase K. (2014). Gut microbiota–generated metabolites in animal health and disease. Nat Chem Biol.

[20] Abrams G.D., Bishop J.E. (1967). Effect of the normal microbial flora on gastrointestinal motility. Proc Soc Exp Biol Med.

[21] Gustafsson B.E., Midtvedt T., Strandberg K. (1970). Effects of microbial contamination on the cecum enlargement of germfree rats. Scand J Gastroenterol.

[22] Anitha M., Vijay-Kumar M., Sitaraman S.V. (2012). Gut microbial products regulate murine gastrointestinal motility via Toll-like receptor 4 signaling. Gastroenterology.

[23] McVey Neufeld K.A., Mao Y.K., Bienenstock J. (2012). The microbiome is essential for normal gut intrinsic primary afferent neuron excitability in the mouse. Neurogastroenterol Motil.

[24] Perez-Burgos A., Perez-Burgos A., Mao Y.-K. (2014). The gut-brain axis rewired: adding a functional vagal nicotinic “sensory synapse”. FASEB J.

[25] Kabouridis P.S., Lasrado R., McCallum S. (2015). Microbiota controls the homeostasis of glial cells in the gut lamina propria. Neuron.

[26] Kashyap P.C., Marcobal A., Ursell L.K. (2013). Complex interactions among diet, gastrointestinal transit, and gut microbiota in humanized mice. Gastroenterology.

[27] Collins J., Borojevic R., Verdu E.F. (2013). Intestinal microbiota influence the early postnatal development of the enteric nervous system. Neurogastroenterol Motil.

[28] Deshmukh H.S., Liu Y., Menkiti O.R. (2014). The microbiota regulates neutrophil homeostasis and host resistance to Escherichia coli K1 sepsis in neonatal mice. Nat Med.

[29] Ardissone A.N., la Cruz de D.M., Davis-Richardson A.G. (2014). Meconium microbiome analysis identifies bacteria correlated with premature birth. PLoS One.

[30] Jiménez E., Marín M.L., Martín R. (2008). Is meconium from healthy newborns actually sterile?. Res Microbiol.

[31] Thorburn A.N., McKenzie C.I., Shen S. (2015). Evidence that asthma is a developmental origin disease influenced by maternal diet and bacterial metabolites. Nat Commun.

[32] Hsiao E.Y., McBride S.W., Hsien S. (2013). Microbiota modulate behavioral and physiological abnormalities associated with neurodevelopmental disorders. Cell.

[33] Choi G.B., Choi G.B., Yim Y.S. (2016). The maternal interleukin-17a pathway in mice promotes autism-like phenotypes in offspring. Science.

[34] Gomez de Agüero M., Ganal-Vonarburg S.C., Fuhrer T. (2016). The maternal microbiota drives early postnatal innate immune development. Science.

[35] Brun P., Giron M.C., Qesari M. (2013). Toll-like receptor 2 regulates intestinal inflammation by controlling integrity of the enteric nervous system. Gastroenterology.

[36] Turco F., Sarnelli G., Cirillo C. (2013). Enteroglial-derived S100B protein integrates bacteria-induced Toll-like receptor signalling in human enteric glial cells. Gut.

[37] Walters K.-A., Olsufka R., Kuestner R.E. (2015). Prior infection with type A Francisella tularensis antagonizes the pulmonary transcriptional response to an aerosolized Toll-like receptor 4 agonist. BMC Genomics.

[38] Wu S.-C., Rau C.-S., Lu T.-H. (2013). Knockout of TLR4 and TLR2 impair the nerve regeneration by delayed demyelination but not remyelination. J Biomed Sci.

[39] Furusawa Y., Obata Y., Fukuda S. (2013). Commensal microbe-derived butyrate induces the differentiation of colonic regulatory T cells. Nature.

[40] Trompette A., Gollwitzer E.S., Yadava K. (2014). Gut microbiota metabolism of dietary fiber influences allergic airway disease and hematopoiesis. Nat Med.

[41] Erny D., Hrabě de Angelis A.L., Jaitin D. (2015). Host microbiota constantly control maturation and function of microglia in the CNS. Nat Neurosci.

[42] Braniste V., Braniste V., Al-Asmakh M. (2014). The gut microbiota influences blood-brain barrier permeability in mice. Sci Transl Med.

[43] Yano J.M., Yu K., Donaldson G.P. (2015). Indigenous bacteria from the gut microbiota regulate host serotonin biosynthesis. Cell.

[44] Reigstad C.S., Reigstad C.S., Salmonson C.E. (2015). Gut microbes promote colonic serotonin production through an effect of short-chain fatty acids on enterochromaffin cells. FASEB J.

[45] Gershon M.D., Tack J. (2007). The serotonin signaling system: from basic understanding to drug development for functional GI disorders. Gastroenterology.

[46] Mercado C.P., Quintero M.V., Li Y. (2013). A serotonin-induced N-glycan switch regulates platelet aggregation. Sci Rep.

[47] Atarashi K., Tanoue T., Oshima K. (2013). Treg induction by a rationally selected mixture of Clostridia strains from the human microbiota. Nature.

[48] Bian X., Patel B., Dai X. (2007). High mucosal serotonin availability in neonatal guinea pig ileum is associated with low serotonin transporter expression. Gastroenterology.

[49] Liu M.-T., Kuan Y.-H., Wang J. (2009). 5-HT4 receptor-mediated neuroprotection and neurogenesis in the enteric nervous system of adult mice. J Neurosci.

[50] Mendoza C., Matheus N., Iceta R. (2009). Lipopolysaccharide induces alteration of serotonin transporter in human intestinal epithelial cells. Innate Immun.

[51] Soret R., Chevalier J., De Coppet P. (2010). Short-chain fatty acids regulate the enteric neurons and control gastrointestinal motility in rats. Gastroenterology.

[52] Moreau N.M., Champ M.M., Goupry S.M. (2004). Resistant starch modulates in vivo colonic butyrate uptake and its oxidation in rats with dextran sulfate sodium-induced colitis. J Nutr.

[53] Cossais F., Durand T., Chevalier J. (2016). Postnatal development of the myenteric glial network and its modulation by butyrate. Am J Physiol Gastrointest Liver Physiol.

[54] Tang C., Ahmed K., Gille A. (2015). Loss of FFA2 and FFA3 increases insulin secretion and improves glucose tolerance in type 2 diabetes. Nat Med.

[55] Nøhr M.K., Pedersen M.H., Gille A. (2013). GPR41/FFAR3 and GPR43/FFAR2 as cosensors for short-chain fatty acids in enteroendocrine cells vs FFAR3 in enteric neurons and FFAR2 in enteric leukocytes. Endocrinology.

[56] Tolhurst G., Tolhurst G., Heffron H. (2012). Short-chain fatty acids stimulate glucagon-like peptide-1 secretion via the g-protein-coupled receptor FFAR2. Diabetes.

[57] Marathe C.S., Rayner C.K., Jones K.L. (2011). Effects of GLP-1 and incretin-based therapies on gastrointestinal motor function. Exp Diabetes Res.

[58] Zhou J., Zhou J., Martin R.J. (2008). Dietary resistant starch upregulates total GLP-1 and PYY in a sustained day-long manner through fermentation in rodents. Am J Physiol Endocrinol Metab.

[59] Breton J., Tennoune N., Lucas N. (2016). Gut commensal E. coli proteins activate host satiety pathways following nutrient-induced bacterial growth. Cell Metab.

[60] Panaro B.L., Tough I.R., Engelstoft M.S. (2014). The melanocortin-4 receptor is expressed in enteroendocrine L cells and regulates the release of peptide YY and glucagon-like peptide 1 in vivo. Cell Metab.

[61] Wichmann A., Allahyar A., Greiner T.U. (2013). Microbial modulation of energy availability in the colon regulates intestinal transit. Cell Host Microbe.

[62] Candido E.P., Reeves R., Davie J.R. (1978). Sodium butyrate inhibits histone deacetylation in cultured cells. Cell.

[63] Davie J.R. (2003). Inhibition of histone deacetylase activity by butyrate. J Nutr.

[64] Falkenberg K.J., Johnstone R.W. (2014). Histone deacetylases and their inhibitors in cancer, neurological diseases and immune disorders. Nat Rev Drug Discov.

[65] Arpaia N., Campbell C., Fan X. (2013). Metabolites produced by commensal bacteria promote peripheral regulatory T-cell generation. Nature.

[66] Chang P.V., Hao L., Offermanns S. (2014). The microbial metabolite butyrate regulates intestinal macrophage function via histone deacetylase inhibition. Proc Natl Acad Sci U S A.

[67] Poole D.P., Godfrey C., Cattaruzza F. (2010). Expression and function of the bile acid receptor GpBAR1 (TGR5) in the murine enteric nervous system. Neurogastroenterol Motil.

[68] Alemi F., Poole D.P., Chiu J. (2013). The receptor TGR5 mediates the prokinetic actions of intestinal bile acids and is required for normal defecation in mice. Gastroenterology.

[69] Dey N., Wagner V.E., Blanton L.V. (2015). Regulators of gut motility revealed by a gnotobiotic model of diet-microbiome interactions related to travel. Cell.

[70] Mikkelsen H.B., Rumessen J.J. (1992). Characterization of macrophage-like cells in the external layers of human small and large intestine. Cell Tissue Res.

[71] Muller P.A., Koscsó B., Rajani G.M. (2014). Crosstalk between muscularis macrophages and enteric neurons regulates gastrointestinal motility. Cell.

[72] Chalazonitis A., Chalazonitis A., D'Autréaux F. (2004). Bone morphogenetic protein-2 and -4 limit the number of enteric neurons but promote development of a TrkC-expressing neurotrophin-3-dependent subset. J Neurosci.

[73] Chalazonitis A., Pham T.D., Li Z. (2008). Bone morphogenetic protein regulation of enteric neuronal phenotypic diversity: relationship to timing of cell cycle exit. J Comp Neurol.

[74] Gabanyi I., Muller P.A., Feighery L. (2016). Neuro-immune interactions drive tissue programming in intestinal macrophages. Cell.

[75] de Jonge W.J. (2013). The gut’s little brain in control of intestinal immunity. ISRN Gastroenterol.

[76] Heanue T.A., Pachnis V. (2007). Enteric nervous system development and Hirschsprung's disease: advances in genetic and stem cell studies. Nat Rev Neurosci.

[77] Enck P., Aziz Q., Barbara G. (2016). Irritable bowel syndrome. Nat Rev Dis Primers.

[78] Kennedy P.J., Cryan J.F., Dinan T.G. (2014). Irritable bowel syndrome: a microbiome-gut-brain axis disorder?. World J Gastroenterol.

[79] Collins S.M. (2014). A role for the gut microbiota in IBS. Nat Rev Gastroenterol Hepatol.

[80] Klingelhoefer L., Reichmann H. (2015). Pathogenesis of Parkinson disease—the gut–brain axis and environmental factors. Nat Rev Neurol.

[81] Michel P.P., Hirsch E.C., Hunot S. (2016). Understanding dopaminergic cell death pathways in Parkinson disease. Neuron.

[82] Fasano A., Visanji N.P., Liu L.W. (2015). Gastrointestinal dysfunction in Parkinson's disease. Lancet Neurol.

[83] Shannon K.M., Keshavarzian A., Dodiya H.B. (2012). Is alpha-synuclein in the colon a biomarker for premotor Parkinson's disease? Evidence from 3 cases. Mov Disord.

[84] Svensson E., Horváth-Puhó E., Thomsen R.W. (2015). Vagotomy and subsequent risk of Parkinson's disease. Ann Neurol.

[85] Holmqvist S., Chutna O., Bousset L. (2014). Direct evidence of Parkinson pathology spread from the gastrointestinal tract to the brain in rats. Acta Neuropathol.

[86] Scheperjans F., Aho V., Pereira P.A.B. (2014). Gut microbiota are related to Parkinson's disease and clinical phenotype. Mov Disord.

[87] Devos D., Lebouvier T., Lardeux B. (2013). Colonic inflammation in Parkinson's disease. Neurobiol Dis.

[88] Clairembault T., Kamphuis W., Leclair-Visonneau L. (2014). Enteric GFAP expression and phosphorylation in Parkinson's disease. J Neurochem.

[89] Brimberg L., Mader S., Fujieda Y. (2015). Antibodies as mediators of brain pathology. Trends Immunol.

[90] Perry R.J., Peng L., Barry N.A. (2016). Acetate mediates a microbiome–brain–β-cell axis to promote metabolic syndrome. Nature.

[91] Frost G., Sleeth M.L., Sahuri-Arisoylu M. (2014). The short-chain fatty acid acetate reduces appetite via a central homeostatic mechanism. Nat Commun.

[92] Buffington S.A., Di Prisco G.V., Auchtung T.A. (2016). Microbial reconstitution reverses maternal diet-induced social and synaptic deficits in offspring. Cell.

[93] Benakis C., Brea D., Caballero S. (2016). Commensal microbiota affects ischemic stroke outcome by regulating intestinal γδ T cells. Nat Med.

[94] Bercik P., Park A.J., Sinclair D. (2011). The anxiolytic effect of Bifidobacterium longum NCC3001 involves vagal pathways for gut-brain communication. Neurogastroenterol Motil.

[95] Bravo J.A., Forsythe P., Chew M.V. (2011). Ingestion of Lactobacillus strain regulates emotional behavior and central GABA receptor expression in a mouse via the vagus nerve. Proc Natl Acad Sci U S A.

